# Methyl 2-oxo-2*H*-chromene-3-carboxyl­ate

**DOI:** 10.1107/S1600536812040044

**Published:** 2012-09-26

**Authors:** Aamer Saeed, Aalia Ibrar, Muhammad Arshad, Michael Bolte

**Affiliations:** a Department of Chemistry, Quaid-i-Azam University, Islamabad 45320, Pakistan; b Chemistry Division, Directorate of Science, PINSTECH, Nilore, Islamabad, Pakistan; c Institut für Anorganische Chemie, J. W. Goethe-Universität Frankfurt, Max-von-Laue-Strasse 7, 60438 Frankfurt/Main, Germany.

## Abstract

The title compound, C_11_H_8_O_4_, features an almost planar mol­ecule (r.m.s. deviation = 0.033 Å for all non-H atoms). In the crystal, the mol­ecules are linked *via* C—H⋯O hydrogen bonds, forming two-dimensional networks lying parallel to (1-21).

## Related literature

For details of the biological activity of coumarins, see: Surya *et al.* (2006 ▶); Kostova (2006 ▶); Reddy *et al.* (2002 ▶); Lacy & O’Kennedy (2004 ▶). For other applications of coumarins, see: Flašík *et al.* (2009 ▶); Fonsecaa *et al.* (2010 ▶).
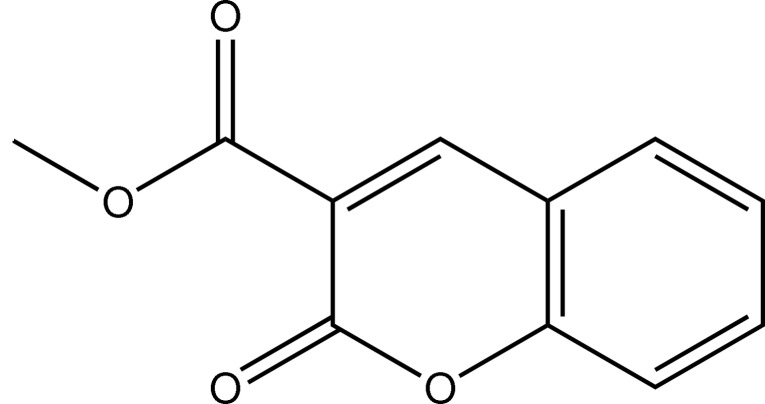


## Experimental

### 

#### Crystal data

C_11_H_8_O_4_
*M* *_r_* = 204.17
Triclinic, 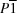

*a* = 3.8874 (10) Å
*b* = 9.782 (3) Å
*c* = 13.078 (3) Å
α = 111.569 (19)°
β = 90.83 (2)°
γ = 95.01 (2)°
*V* = 460.1 (2) Å^3^
*Z* = 2
Mo *K*α radiation
μ = 0.11 mm^−1^
*T* = 173 K
0.30 × 0.27 × 0.20 mm

#### Data collection

Stoe IPDS II two-circle diffractometer
Absorption correction: multi-scan (*X-RED32*; Stoe & Cie, 2001 ▶) *T*_min_ = 0.967, *T*_max_ = 0.978
4851 measured reflections
1725 independent reflections
1378 reflections with *I* > 2σ(*I*)
*R*_int_ = 0.054

#### Refinement

*R*[*F* ^2^ > 2σ(*F* ^2^)] = 0.043
*wR*(*F* ^2^) = 0.123
*S* = 1.09
1725 reflections
136 parameters
H-atom parameters constrained
Δρ_max_ = 0.21 e Å^−3^
Δρ_min_ = −0.18 e Å^−3^

### 

Data collection: *X-AREA* (Stoe & Cie, 2001 ▶); cell refinement: *X-AREA*; data reduction: *X-RED32* (Stoe & Cie, 2001 ▶); program(s) used to solve structure: *SHELXS97* (Sheldrick, 2008 ▶); program(s) used to refine structure: *SHELXL97* (Sheldrick, 2008 ▶); molecular graphics: *XP* in *SHELXTL-Plus* (Sheldrick, 2008 ▶); software used to prepare material for publication: *SHELXL97*.

## Supplementary Material

Crystal structure: contains datablock(s) global, I. DOI: 10.1107/S1600536812040044/su2501sup1.cif

Structure factors: contains datablock(s) I. DOI: 10.1107/S1600536812040044/su2501Isup2.hkl

Supplementary material file. DOI: 10.1107/S1600536812040044/su2501Isup3.mol

Supplementary material file. DOI: 10.1107/S1600536812040044/su2501Isup4.cml

Additional supplementary materials:  crystallographic information; 3D view; checkCIF report

## Figures and Tables

**Table 1:** Hydrogen-bond geometry (Å, °)

*D*—H⋯*A*	*D*—H	H⋯*A*	*D*⋯*A*	*D*—H⋯*A*
C3—H3⋯O3^i^	0.95	2.54	3.360 (2)	145
C5—H5⋯O3^i^	0.95	2.46	3.298 (2)	147
C8—H8⋯O1^ii^	0.95	2.55	3.454 (2)	160
C11—H11*A*⋯O2^iii^	0.98	2.53	3.354 (2)	142

Symmetry codes: (i) 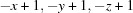; (ii) 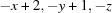; (iii) 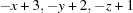.

**Fig. 1.**
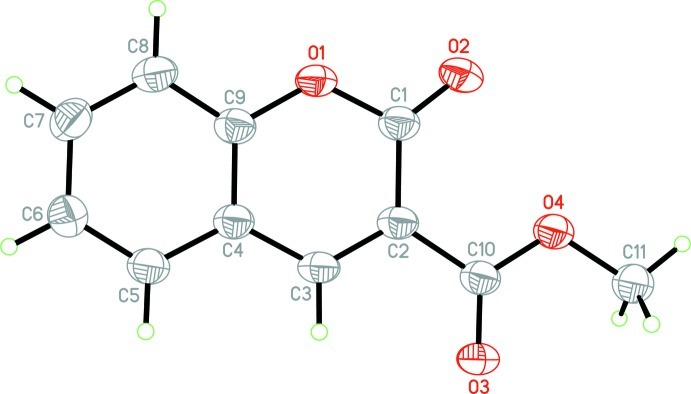
Molecular structure of title molecule, with the atom numbering. Displacement ellipsoids are drawn at the 50% probability level.

**Fig. 2.**
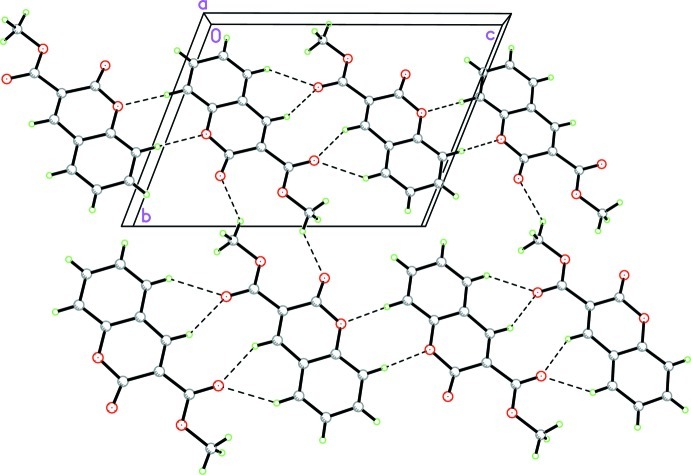
Crystal packing of the title compound view along [100]. Hydrogen bonds are drawn as dashed lines.

## supplementary crystallographic information

### Comment

Coumarins (2*H*-1-benzopyran-2-ones) are natural lactones and amongst the best known oxygen heterocycles, well represented as a structural motif in numerous natural products (Surya *et al.*, 2006). Various coumarin derivatives are known to possess an array of biological activities, including anticancer, anti-HIV, anti acetylcholinesterase, antifungal, antioxidant, antihelmintic, anticoagulant, antibacterial, antiviral and anticlotting activities, and find extensive application in pharmaceuticals, fragrances, agrochemicals, additives in food and cosmetics and insecticides (Kostova, 2006; Reddy *et al.*, 2002; Lacy & O'Kennedy, 2004). Moreover, coumarins find applications as dyes in laser technology, fluorescent indicators, optical brighteners and photosensitizers (Flašík *et al.*, 2009). Ethyl 2-oxo-2H-chromene-3-carboxylate irreversibly inhibits phospholipase A2 (sPLA2) from Crotalus durissus ruruima venom with an IC50 of 3.1 ± 0.06 nmol (Fonsecaa *et al.*, 2010).

The title compound, Fig. 1, features an almost planar molecule (r.m.s. deviation = 0.033 Å for all non-H atoms). The maximum deviation from the mean plane being 0.0734 (12) Å for atom O2.

In the crystal, molecules are linked via C—H···O hydrogen bonds forming two-dimensional networks lying parallel to (121); Table 1 and Fig. 2.

### Experimental

Salicylaldehyde (1.22 g, 0.01 mol) and diethylmalonate (1.6 g, 0.01 mol) were dissolved in ethanol to give a clear solution. Piperidine (2 ml) was added and the mixture was refluxed for 5 h. The content was concentrated to a small volume. The product (3) was poured onto crushed ice, filtered out and crystallized from ethanol to give colourless crystals, m.p. 393–395 K; Yield: 90%. Spectroscopic data for the title compound are available in the archived CIF.

### Refinement

All the H atoms were included in calculated positions and treated as riding atoms: C_aromatic_—H = 0.95 Å and C_methyl_—H = 0.98 Å, with *U*_iso_(H) = 1.2*U*_eq_(C) or *U*_iso_(H) = 1.5*U*_eq_(C_methyl_).

### Crystal data

**Table:**

C_11_H_8_O_4_	*Z* = 2
*M**_r_* = 204.17	*F*(000) = 212
Triclinic, *P*1	*D*_x_ = 1.474 Mg m^−^^3^
Hall symbol: -P 1	Mo *K*α radiation, λ = 0.71073 Å
*a* = 3.8874 (10) Å	Cell parameters from 10306 reflections
*b* = 9.782 (3) Å	θ = 3.3–26.0°
*c* = 13.078 (3) Å	µ = 0.11 mm^−^^1^
α = 111.569 (19)°	*T* = 173 K
β = 90.83 (2)°	Block, colourless
γ = 95.01 (2)°	0.30 × 0.27 × 0.20 mm
*V* = 460.1 (2) Å^3^	

### Data collection

**Table:**

Stoe IPDS II two-circle diffractometer	1725 independent reflections
Radiation source: Genix 3D IµS microfocus X-ray source	1378 reflections with *I* > 2σ(*I*)
Genix 3D multilayer optics monochromator	*R*_int_ = 0.054
ω scans	θ_max_ = 25.7°, θ_min_ = 4.2°
Absorption correction: multi-scan (*X-RED32*; Stoe & Cie, 2001)	*h* = −4→4
*T*_min_ = 0.967, *T*_max_ = 0.978	*k* = −11→11
4851 measured reflections	*l* = −15→15

### Refinement

**Table:**

Refinement on *F*^2^	Primary atom site location: structure-invariant direct methods
Least-squares matrix: full	Secondary atom site location: difference Fourier map
*R*[*F*^2^ > 2σ(*F*^2^)] = 0.043	Hydrogen site location: inferred from neighbouring sites
*wR*(*F*^2^) = 0.123	H-atom parameters constrained
*S* = 1.09	*w* = 1/[σ^2^(*F*_o_^2^) + (0.0591*P*)^2^ + 0.0733*P*] where *P* = (*F*_o_^2^ + 2*F*_c_^2^)/3
1725 reflections	(Δ/σ)_max_ < 0.001
136 parameters	Δρ_max_ = 0.21 e Å^−^^3^
0 restraints	Δρ_min_ = −0.18 e Å^−^^3^

### Special details

**Table:**

Experimental. Spectroscopic data for the title compound: IR (KBr, cm^-1^) 1710 (C═O, coumarin), 1670 (C═O), 1750 (C═O, ester), 1200 (C—O); ^1^HNMR (DMSO-d6, 300MHz, δ p.p.m.): 7.5 (4H, m, Ar—H), 8.1 (1H, s, Ar—H, H-4), 1.83 (3H, t, CH3), 3.20 (2H, q, CH2). Mass m/z (%): 216 M^+^.
Geometry. All e.s.d.'s (except the e.s.d. in the dihedral angle between two l.s. planes) are estimated using the full covariance matrix. The cell e.s.d.'s are taken into account individually in the estimation of e.s.d.'s in distances, angles and torsion angles; correlations between e.s.d.'s in cell parameters are only used when they are defined by crystal symmetry. An approximate (isotropic) treatment of cell e.s.d.'s is used for estimating e.s.d.'s involving l.s. planes.
Refinement. Refinement of *F*^2^ against ALL reflections. The weighted *R*-factor *wR* and goodness of fit *S* are based on *F*^2^, conventional *R*-factors *R* are based on *F*, with *F* set to zero for negative *F*^2^. The threshold expression of *F*^2^ > σ(*F*^2^) is used only for calculating *R*-factors(gt) *etc*. and is not relevant to the choice of reflections for refinement. *R*-factors based on *F*^2^ are statistically about twice as large as those based on *F*, and *R*-factors based on ALL data will be even larger.

### Fractional atomic coordinates and isotropic or equivalent isotropic displacement parameters (Å^2^)

**Table:**

	*x*	*y*	*z*	*U*_iso_*/*U*_eq_	
O1	0.8969 (3)	0.56201 (13)	0.15727 (9)	0.0371 (3)	
O2	1.2039 (3)	0.76399 (14)	0.26510 (10)	0.0472 (4)	
O3	0.8489 (3)	0.68616 (13)	0.54600 (9)	0.0469 (4)	
O4	1.1674 (3)	0.84024 (13)	0.48731 (9)	0.0394 (3)	
C1	1.0100 (4)	0.66021 (18)	0.26166 (14)	0.0352 (4)	
C2	0.8796 (4)	0.62198 (18)	0.35402 (13)	0.0328 (4)	
C3	0.6775 (4)	0.49482 (18)	0.33393 (13)	0.0329 (4)	
H3	0.5978	0.4715	0.3945	0.039*	
C4	0.5793 (4)	0.39425 (18)	0.22513 (13)	0.0330 (4)	
C5	0.3781 (4)	0.25926 (18)	0.20103 (14)	0.0371 (4)	
H5	0.2943	0.2314	0.2591	0.045*	
C6	0.3016 (4)	0.1670 (2)	0.09335 (15)	0.0408 (4)	
H6	0.1695	0.0745	0.0773	0.049*	
C7	0.4178 (4)	0.2090 (2)	0.00751 (14)	0.0422 (4)	
H7	0.3604	0.1452	−0.0666	0.051*	
C8	0.6144 (4)	0.34155 (19)	0.02878 (14)	0.0385 (4)	
H8	0.6927	0.3700	−0.0296	0.046*	
C9	0.6949 (4)	0.43218 (18)	0.13747 (13)	0.0338 (4)	
C10	0.9625 (4)	0.71847 (18)	0.47158 (13)	0.0338 (4)	
C11	1.2460 (4)	0.9364 (2)	0.60135 (14)	0.0415 (4)	
H11A	1.3986	1.0231	0.6043	0.062*	
H11B	1.3614	0.8827	0.6399	0.062*	
H11C	1.0311	0.9682	0.6368	0.062*	

### Atomic displacement parameters (Å^2^)

**Table:**

	*U*^11^	*U*^22^	*U*^33^	*U*^12^	*U*^13^	*U*^23^
O1	0.0401 (6)	0.0439 (7)	0.0297 (6)	−0.0047 (5)	0.0051 (5)	0.0184 (5)
O2	0.0525 (7)	0.0500 (7)	0.0411 (7)	−0.0127 (6)	0.0077 (6)	0.0228 (6)
O3	0.0582 (7)	0.0504 (8)	0.0306 (7)	−0.0130 (6)	0.0077 (5)	0.0171 (6)
O4	0.0420 (6)	0.0437 (7)	0.0321 (6)	−0.0096 (5)	−0.0006 (5)	0.0167 (5)
C1	0.0333 (8)	0.0419 (9)	0.0325 (9)	−0.0004 (7)	0.0049 (6)	0.0171 (7)
C2	0.0294 (7)	0.0411 (9)	0.0312 (9)	0.0011 (6)	0.0042 (6)	0.0178 (7)
C3	0.0309 (8)	0.0423 (9)	0.0301 (8)	0.0021 (7)	0.0053 (6)	0.0191 (7)
C4	0.0304 (7)	0.0400 (9)	0.0313 (9)	0.0015 (6)	0.0033 (6)	0.0165 (7)
C5	0.0349 (8)	0.0450 (9)	0.0348 (9)	−0.0009 (7)	0.0024 (7)	0.0198 (8)
C6	0.0395 (8)	0.0422 (9)	0.0401 (10)	−0.0031 (7)	−0.0016 (7)	0.0161 (8)
C7	0.0407 (9)	0.0506 (10)	0.0318 (9)	0.0017 (8)	−0.0016 (7)	0.0120 (8)
C8	0.0372 (8)	0.0505 (10)	0.0318 (9)	0.0043 (7)	0.0044 (7)	0.0199 (8)
C9	0.0300 (7)	0.0407 (9)	0.0339 (9)	0.0018 (6)	0.0034 (6)	0.0179 (7)
C10	0.0319 (8)	0.0390 (9)	0.0333 (9)	−0.0003 (6)	0.0032 (6)	0.0173 (7)
C11	0.0417 (9)	0.0456 (10)	0.0356 (9)	−0.0061 (7)	−0.0029 (7)	0.0158 (8)

### Geometric parameters (Å, º)

**Table:**

O1—C9	1.3691 (19)	C4—C5	1.400 (2)
O1—C1	1.387 (2)	C5—C6	1.376 (2)
O2—C1	1.1970 (19)	C5—H5	0.9500
O3—C10	1.2069 (19)	C6—C7	1.398 (3)
O4—C10	1.3209 (19)	C6—H6	0.9500
O4—C11	1.452 (2)	C7—C8	1.378 (2)
C1—C2	1.474 (2)	C7—H7	0.9500
C2—C3	1.348 (2)	C8—C9	1.384 (2)
C2—C10	1.491 (2)	C8—H8	0.9500
C3—C4	1.424 (2)	C11—H11A	0.9800
C3—H3	0.9500	C11—H11B	0.9800
C4—C9	1.397 (2)	C11—H11C	0.9800
			
C9—O1—C1	123.86 (12)	C7—C6—H6	119.9
C10—O4—C11	115.67 (12)	C8—C7—C6	120.97 (17)
O2—C1—O1	115.90 (14)	C8—C7—H7	119.5
O2—C1—C2	128.38 (16)	C6—C7—H7	119.5
O1—C1—C2	115.71 (14)	C7—C8—C9	118.30 (15)
C3—C2—C1	120.00 (16)	C7—C8—H8	120.9
C3—C2—C10	117.20 (14)	C9—C8—H8	120.9
C1—C2—C10	122.80 (15)	O1—C9—C8	117.63 (13)
C2—C3—C4	122.29 (14)	O1—C9—C4	120.23 (15)
C2—C3—H3	118.9	C8—C9—C4	122.14 (15)
C4—C3—H3	118.9	O3—C10—O4	123.18 (16)
C9—C4—C5	118.29 (15)	O3—C10—C2	121.75 (15)
C9—C4—C3	117.80 (15)	O4—C10—C2	115.07 (13)
C5—C4—C3	123.90 (14)	O4—C11—H11A	109.5
C6—C5—C4	120.13 (15)	O4—C11—H11B	109.5
C6—C5—H5	119.9	H11A—C11—H11B	109.5
C4—C5—H5	119.9	O4—C11—H11C	109.5
C5—C6—C7	120.15 (16)	H11A—C11—H11C	109.5
C5—C6—H6	119.9	H11B—C11—H11C	109.5
			
C9—O1—C1—O2	175.14 (13)	C1—O1—C9—C8	−177.09 (13)
C9—O1—C1—C2	−4.0 (2)	C1—O1—C9—C4	2.4 (2)
O2—C1—C2—C3	−175.95 (16)	C7—C8—C9—O1	178.57 (13)
O1—C1—C2—C3	3.1 (2)	C7—C8—C9—C4	−0.9 (2)
O2—C1—C2—C10	3.8 (3)	C5—C4—C9—O1	−178.95 (13)
O1—C1—C2—C10	−177.24 (12)	C3—C4—C9—O1	0.3 (2)
C1—C2—C3—C4	−0.7 (2)	C5—C4—C9—C8	0.6 (2)
C10—C2—C3—C4	179.63 (13)	C3—C4—C9—C8	179.76 (13)
C2—C3—C4—C9	−1.1 (2)	C11—O4—C10—O3	−0.9 (2)
C2—C3—C4—C5	178.10 (14)	C11—O4—C10—C2	179.09 (12)
C9—C4—C5—C6	0.7 (2)	C3—C2—C10—O3	−1.4 (2)
C3—C4—C5—C6	−178.50 (14)	C1—C2—C10—O3	178.88 (14)
C4—C5—C6—C7	−1.4 (2)	C3—C2—C10—O4	178.57 (13)
C5—C6—C7—C8	1.0 (2)	C1—C2—C10—O4	−1.1 (2)
C6—C7—C8—C9	0.1 (2)		

### Hydrogen-bond geometry (Å, º)

**Table:**

*D*—H···*A*	*D*—H	H···*A*	*D*···*A*	*D*—H···*A*
C3—H3···O3^i^	0.95	2.54	3.360 (2)	145
C5—H5···O3^i^	0.95	2.46	3.298 (2)	147
C8—H8···O1^ii^	0.95	2.55	3.454 (2)	160
C11—H11*A*···O2^iii^	0.98	2.53	3.354 (2)	142

Symmetry codes: (i) −*x*+1, −*y*+1, −*z*+1; (ii) −*x*+2, −*y*+1, −*z*; (iii) −*x*+3, −*y*+2, −*z*+1.
